# Supplementary material for: HPV18 E7 inhibits LATS1 kinase and activates YAP1 by degrading PTPN14
Source: bioRxiv. 2024 Jun 19:2024.03.07.583953. Originally published 2024 Mar 7. Preprint. [Version 2] doi: 10.1101/2024.03.07.583953 (PMC10942435; doi:10.1101/2024.03.07.583953)
Supplement: Supplement 1 — Supplementary Table 1. Densitometry analysis of western blots in Figures 1, 2, 3, and 8. [file media-1.pdf]

Figure 1

	sgNT-1							
CytoD (min):	0	backgroun	30	backgroun	60	backgroun	90	backgroun
pYAP	95.303	44.068	134.949	49.318	129.412	49.333	149.736	60.071
YAP	65.683	30.969	74.743	34.251	66.27	30.603	66.386	30.608
pYAP	92.578	33.268	105.273	36.367	112.567	36.308	113.064	36.671
YAP	83.408	55.021	82.099	47.664	71.585	37.286	71.832	40.654
pYAP	110.828	53.521	133.436	58.713	133.952	53.404	160.787	56.62
YAP	143.04	117.467	130.703	83.014	123.591	71.905	124.772	80.489
	sgNT-2							
CytoD (min):	0	backgroun	30	backgroun	60	backgroun	90	backgroun
pYAP	98.969	50.187	117.255	53.26	116.279	53.82	141.542	53.976
YAP	65.524	32.752	66.395	30.681	64.994	35.158	65.891	32.897
pYAP	96.686	35.882	94.666	40.016	103.062	38.516	128.11	41.205
YAP	73.554	40.901	75.527	44.527	73.508	40.581	77.888	40.478
pYAP	134.693	56.284	154.433	60.031	143.515	57.859	152.316	58.997
YAP	138.742	96.147	145.314	104.091	135.373	91.89	129.71	92.697
	sgNT-1				sgPTPN14-1			
CytoD (min):	0	30	60	90	0	30	60	90
pYAP/YAP	1.475917	2.114763	2.245185	2.506149	1.375056	1.540785	1.859305	1.817944
pYAP/YAP	2.089337	2.001045	2.223359	2.450221	1.146721	1.04133	1.001112	1.240461
pYAP/YAP	2.240918	1.566881	1.55841	2.352302	1.020363	1.533598	1.769807	1.981521

Figure 2A

	Untreated				Suspension			
	sgNT-1	backgroun	sgPTPN14-	backgroun	sgNT-1	backgroun	sgPTPN14-	backgroun
pYAP	68.777	36.141	65.497	36.858	129.537	48.027	99.97	39.908
YAP	102.93	76.023	98.933	72.108	99.841	71.113	102.535	73.171
pLATS1	117.085	105.495	119.055	115.555	172.38	123.538	161.298	147.89
LATS1	177.479	130.268	177.602	129.867	181.014	130.426	180.368	130.209
pNF2	80.429	26.493	86.925	27.636	57.142	28.366	57.151	25.617
NF2	78.661	18.603	72.345	11.8	89.663	11.867	87.679	15.401
pYAP	48.776	34.169	44.075	34.448	209.361	70.576	60.789	38.491
YAP	88.585	68.323	83.202	66.928	112.486	76.188	77.182	67.471
pLATS1	134.411	134.371	134.864	133.557	233.918	166.639	193.77	183.397
LATS1	162.84	129.07	155.812	127.962	206.061	134.651	154.185	127.467
pNF2	207.226	177.823	201.37	179.366	206.513	183.535	182.262	174.584
NF2	181.947	115.065	162.294	108.574	195.134	117.7	172.227	103.363
pYAP	75.971	35.997	51.796	31.42	177.681	42.938	90.313	40.183
YAP	77.429	44.703	65.529	44.282	91.283	49.26	69.505	47.77
pLATS1	122.779	119.771	129.302	124.946	183.826	140.474	144.712	129.446
LATS1	123.064	52.671	91.275	54.371	145.509	65.703	106.192	55.044
pNF2	169.283	82.455	145.24	75.146	132.314	76.225	102.362	74.883
NF2	119.222	83.063	128.167	80.773	171.173	85.8	149.321	81.707

Figure 2B

	Untreated				Suspension			
	sgNT-2	backgroun	sgPTPN14-	backgroun	sgNT-2	backgroun	sgPTPN14-	backgroun
pYAP	131.684	83.432	107.042	70.532	186.111	68.577	135.365	74.66
YAP	104.428	73.547	92.393	68.102	105.909	67.644	102.016	67.198
pLATS1	126.382	113.68	114.615	108.617	143.861	108.501	118.14	109.903
LATS1	201.244	136.797	197.171	134.16	207.006	136.135	198.907	137.947
pNF2	148.377	77.516	138.814	82.179	117.564	86.121	117.581	89.61
NF2	155.092	81.208	152.42	77.268	164.197	78.586	162.285	73.538
pYAP	133.808	53.184	113.607	46.548	183.054	57.795	140.797	55.937
YAP	90.483	55.763	88.73	52.381	80.199	49.266	89.905	56.373
pLATS1	100.996	90.073	101.114	96.722	139.553	102.692	121.551	103.571
LATS1	198.289	118.126	194.281	114.444	191.537	115.274	195.622	113.453
pNF2	101.861	43.413	108.409	48.409	76.525	45.683	75.262	42.641
NF2	87.821	21.927	79.863	24.584	86.839	25.463	93.321	25.744
pYAP	75.306	37.444	48.099	32.911	147.176	46.011	105.255	36.057
YAP	73.508	45.927	64.675	44.526	72.335	43.658	78.554	46.232
pLATS1	135.985	127.792	116.306	114.322	171.838	112.807	137.974	120.685
LATS1	122.187	57.354	98.016	52.697	135.16	55.751	131.619	61.279
pNF2	161.224	82.187	147.608	75.781	106.046	73.158	112.563	75.783
NF2	145.801	88.374	137.892	81.288	158.71	79.408	163.599	80.133

	Figure 2A				Figure 2B			
	Untreated		Suspension		Untreated		Suspension	
	sgNT-1	sgPTPN14-	sgNT-1	sgPTPN14-	sgNT-2	sgPTPN14-	sgNT-2	sgPTPN14-
pYAP/YAP	1.212919	1.067623	2.837302	2.04543	1.562514	1.503026	3.07158	1.743495
pLATS1/LATS	0.245494	0.073321	0.965486	0.26731	0.197092	0.09519	0.498935	0.135121
pNF2/NF2	0.898065	0.979255	0.36989	0.436288	0.959085	0.753606	0.367278	0.315177
pYAP/YAP	0.720906	0.591557	3.823489	2.296159	2.32212	1.844865	4.049365	2.530717
pLATS1/LATS	0.001184	0.04693	0.942151	0.38824	0.13626	0.055012	0.483341	0.218817
pNF2/NF2	0.439625	0.409605	0.296743	0.111495	0.887	1.085403	0.502509	0.482723
pYAP/YAP	1.221475	0.959006	3.206411	2.306418	1.372757	0.753784	3.52774	2.140895
pLATS1/LATS	0.042732	0.118036	0.543217	0.298467	0.126371	0.043779	0.743379	0.245792
pNF2/NF2	2.401283	1.478964	0.656988	0.40641	1.376304	1.268939	0.414718	0.440658

	Empty Vector							
CytoD (min):	0	backgroun	30	backgroun	60	backgroun	90	backgroun
pYAP	95.481	36.311	118.148	35.408	178.166	44.2	185.098	40.987
YAP	79.159	35.539	72.925	31.477	87.165	36.819	82.064	32.971
pYAP	115.789	39.913	107.597	39.746	97.508	35.667	108.077	38.324
YAP	53.781	30.465	55.299	31.592	48.995	30.091	45.427	30.249
pYAP	120.326	36.68	149.19	45.818	172.591	49.611	193.235	59.809
YAP	82.946	63.677	85.664	66.419	85.375	66.231	86.72	68.064

	EmptyV				18E7			
CytoD (min):	0	30	60	90	0	30	60	90
pYAP/YAP	1.356488	1.996236	2.660907	2.935469	1.390787	1.439709	1.168746	1.276229
pYAP/YAP	3.254246	2.862066	3.271318	4.595665	1.749755	1.427272	0.820016	1.4532

pYAP/YAP	4.340962	5.371369	6.423945	7.151908	3.426362	2.290689	1.95338	2.536999

Figure 3B

	Untreated							
	Empty	backgroun	18E7	backgroun	18E7 R84S	backgroun	Empty	backgroun
pYAP	88.758	31.213	62.958	31.908	77.066	31.994	171.168	38.742
YAP	71.965	45.84	71.822	41.189	68.447	40.266	72.27	33.117
pLATS1	43.577	40.719	40.943	57.649	50.378	67.831	123.273	71.652
LATS1	127.565	73.201	129.633	73.748	131.786	71.384	131.559	71.03
pYAP	121.936	48.81	177.542	59.118	189.315	61.773	230.115	72.566
YAP	157.376	115.482	183.375	119.619	180.261	121.01	175.731	119.438
pLATS1	127.597	124.749	133.062	141.062	139.366	129.443	177.971	138.619
LATS1	103.572	43.675	138.787	46.139	125.722	44.626	131.561	42.345
pYAP	137.035	58.611	97.15	56.072	134.657	59.447	202.275	65.401
YAP	202.143	136.378	184.162	135.842	186.944	131.027	185.666	132.896
pLATS1	110.387	104.633	107.085	102.99	112.995	106.235	156.259	114.724
LATS1	180.313	87.789	165.151	88.852	163.972	85.367	163.032	90.104

	Untreated			Suspension		
	EmptyV	18E7	18E7 R84S	EmptyV	18E7	18E7 R84S
pYAP/YAP	2.202679	1.013613	1.599375	3.38227	1.75801	3.145487
pLATS1/LATS1	0.052572	-0.29894	-0.28895	0.852831	0.206883	0.922873
pYAP/YAP	1.745501	1.857457	2.152571	2.798732	1.760412	3.321504
pLATS1/LATS1	0.047548	-0.08635	0.122361	0.441087	0.110195	0.561575
pYAP/YAP	1.192488	0.850124	1.345029	2.593784	1.316299	2.981392
pLATS1/LATS1	0.062189	0.05367	0.086	0.569534	0.17562	0.513546

Figure 8

	sgNT-1							
	0	backgroun	30	backgroun	60	backgroun	90	backgroun
pYAP	113.227	83.682	147.77	92.754	145.061	91.637	158.317	96.746
YAP	128.566	96.925	119.956	81.758	102.337	70.51	108.233	64.623
pYAP	127.559	96.142	151.516	99.928	160.08	93.455	164.591	91.648
YAP	161.569	121.889	157.559	129.039	147.884	123.055	144.112	120.825
	sgLATS1/2-1							
	0	backgroun	30	backgroun	60	backgroun	90	backgroun
pYAP	102.003	81.804	131.921	90.272	133.38	90.299	132.855	89.913
YAP	99.885	59.732	111.764	61.122	101.887	54.021	97.292	56.711
pYAP	115.236	90.996	149.673	92.467	147.802	93.137	150.269	95.105
YAP	159.816	128.699	163.897	123.527	160.555	128.842	162.213	123.339

	sgNT-1				sgPTPN14-1			
	0	30	60	90	0	30	60	90
pYAP/YAP	0.933757	1.440285	1.678575	1.411855	0.631365	0.851854	0.973989	1.07125
pYAP/YAP	0.791759	1.808836	2.683354	3.132349	1.064157	1.460521	1.516648	1.615688

sgPTPN14-1							
0	backgroun	30	backgroun	60	backgroun	90	background
92.538	46.486	103.181	48.252	119.552	49.168	116.625	56.162
63.634	30.143	67.683	32.033	69.075	31.22	63.74	30.481
63.536	31.867	70.302	32.836	63.363	31.862	69.715	34.115
64.216	36.599	71.834	35.855	65.764	34.298	66.337	37.638
93.563	50.77	127.824	54.334	149.492	55.894	143.859	55.713
129.187	87.248	137.757	89.837	145.141	92.255	143.328	98.844
sgPTPN14-3							
0	backgroun	30	backgroun	60	backgroun	90	background
80.441	42.689	99.469	44.297	93.607	43.249	101.658	45.206
64.036	34.181	71.187	31.987	67.211	35.057	62.911	34.736
83.453	34.527	68.569	32.617	67.516	34.553	59.507	34.824
71.569	36.997	67.287	32.897	71.08	42.083	66.39	41.411
108.125	53.651	108.735	54.194	123.621	55.727	101.688	51.069
128.311	91.164	129.368	92.057	136.012	91.042	135.131	109.094
sgNT-2				sgPTPN14-3			
0	30	60	90	0	30	60	90
1.488527	1.791874	2.093411	2.653998	1.264512	1.407449	1.56615	2.00362
1.862126	1.762903	1.960276	2.323042	1.415191	1.04542	1.136773	0.98815
1.840803	2.290032	1.969873	2.521249	1.466444	1.461794	1.509762	1.944118

d

d

3

Figure 3A

18E7									
0	backgroun	30	backgroun	60	backgroun	90	backgroun	0	backgroun
131.684	37.97	112.693	34.786	88.179	32.826	101.809	35.283	116.162	37.281
109.109	41.727	85.561	31.448	81.245	33.884	86.335	34.208	79.509	35.254
66.897	32.978	56.254	31.458	52.367	34.284	75.271	36.302	91.747	37.468
49.674	30.289	47.592	30.219	52.839	30.787	57.152	30.336	56.919	32.933
103.65	40.742	93.09	42.239	74.436	37.564	95.035	40.591	111.466	42.384
86.86	68.5	93.048	70.849	90.019	71.143	91.453	69.993	91.697	71.246

18E7 R84S			
0	30	60	90
1.78242	2.239326	2.768147	2.92193
2.262945	1.525043	2.087527	3.159851

3.377928	2.497191	2.924334	4.150392

Suspension			
18E7	backgroun	18E7 R84S	background
94.55	32.276	151.452	37.296
65.443	30.02	71.238	34.946
74.118	61.073	120.633	61.056
133.197	70.142	136.463	71.907
157.94	57.211	211.29	64.443
177.256	120.037	163.122	118.911
149.304	139.959	177.085	140.285
128.6	43.796	109.716	44.186
113.209	57.365	193.126	64.151
180.882	138.457	186.413	143.153
126.602	114.235	155.027	114.064
160.551	90.132	166.78	87.015

sgPTPN14-1							
0	backgroun	30	backgroun	60	backgroun	90	background
108.148	82.418	127.76	86.802	146.453	93.13	142.778	94.726
108.954	68.201	117.535	69.454	120.997	66.25	107.366	62.51
117.822	90.454	138.946	91.926	144.967	91.399	152.264	94.571
149.88	124.162	152.072	119.878	160.661	125.341	158.093	122.385
SV40ST							
0	backgroun	30	backgroun	60	backgroun	90	background
89.902	78.266	115.911	84.392	128.08	87.836	121.006	83.218
93.412	50.293	80.315	49.646	104.592	47.388	105.051	75.542
106.939	89.705	133.043	90.5	134.944	93.265	129.483	91.79
150.837	121.157	159.717	121.805	154.162	119.693	166.791	131.333

sgLATS1/2-1				SV40 ST			
0	30	60	90	0	30	60	90
0.503051	0.82242	0.900033	1.05818	0.269858	1.027715	0.703517	1.280558
0.778995	1.417042	1.723741	1.419046	0.58066	1.122151	1.209173	1.063032

18E7 R84S					
30	backgroun	60	backgroun	90	background
138.238	40.001	127.705	38.698	150.022	40.698
78.477	34.608	69.032	36.878	70.915	33.5
76.636	39.123	79.901	36.303	96.187	36.766
57.098	32.5	57.542	36.657	56.529	37.724
101.056	42.372	104.273	41.084	146.479	43.79
90.729	67.229	87.128	65.52	87.975	63.233
