# Supplementary material for: HPV18 E7 inhibits LATS1 kinase and activates YAP1 by degrading PTPN14
Source: bioRxiv. 2024 Jun 19:2024.03.07.583953. Originally published 2024 Mar 7. Preprint. [Version 2] doi: 10.1101/2024.03.07.583953 (PMC10942435; doi:10.1101/2024.03.07.583953)
Supplement: Supplement 2 — Supplementary Table 2. List of plasmids used in the study. [file media-2.pdf]

Name	Lab ID #	Addgene #	Gene	Promoter	Bacterial Resistance	Selection	Epitope Tag	Tag Location	Source
MSCV-IP N HA only empty v2	7270	163302	N/A	MSCV LTR	Ampicillin	Puromycin	HA	N-terminus	White et al (2014) J. Virol. 88(15):8201-12
MSCV-P C-FlagHA 18E7	6641	35019	HPV18 E7	MSCV LTR	Ampicillin	Puromycin	FlagHA	C-terminus	White et al. (2012) PNAS: 109(5):E260–E267
MSCV-P C-FlagHA 18E7 R84S	8193	163307	HPV18 E7 R84S	MSCV LTR	Ampicillin	Puromycin	FlagHA	C-terminus	Hatterschide et al. (2020) J. Virol. 94:e1024-20
pLIX-402	8201	41394	N/A	TRE	Ampicillin+chloramphenicol	N/A	HA	C-terminus	Addgene
pLIX-PTPN14	8221	221643	PTPN14	TRE	Ampicillin	Puromycin	HA	C-terminus	This study
pLIX-PTPN14 ΔPPXY1/2	8224	221644	PTPN14	TRE	Ampicillin	Puromycin	HA	C-terminus	This study
pLIX-PTPN14 ΔPPXY3/4	8552	221645	PTPN14	TRE	Ampicillin	Puromycin	HA	C-terminus	This study
LentiCas9-blast	7527	52962	spCas9	EFS-NS	Ampicillin	Blasticidin		N-terminus	Addgene
pHAGE-P-CMVt N-HA GFP	6571	N/A	GFP	CMVt	Ampicillin	Puromycin	HA	N-terminus	Galligan et al. (2015) J Proteome Res. 14(2): 953–966.
pHAGE-P-CMVt N-V5 PTPN14	7522	N/A	PTPN14	CMVt	Ampicillin	Puromycin	V5	N-terminus	White et al. (2016) mBio. 7(5):e01530-16
pHAGE-P-CMVt N-V5 PTPN14 ΔPPXY1/2	8215	221646	PTPN14	CMVt	Ampicillin	Puromycin	V5	N-terminus	This study
pHAGE-P-CMVt N-V5 PTPN14 ΔPPXY3/4	8180	221647	PTPN14	CMVt	Ampicillin	Puromycin	V5	N-terminus	This study
pHAGE-P-CMVt N-V5 PTPN14 C1121S	8189	221648	PTPN14	CMVt	Ampicillin	Puromycin	V5	N-terminus	This study

Name	Lab ID #	Addgene #	sgRNA sequence	Promoter	Bacterial Resistance	Selection	Source
LentiCRISPRv2-Neo	8175	98292	N/A	U6	Ampicillin	G418	Addgene
LentiCRISPRv2 Neo sgNT-1	8389	221649	GAGCTCGCCATG	U6	Ampicillin	G418	This study
LentiCRISPRv2 Neo sgNT-2	8390	221650	GGATTGTGGTCC	U6	Ampicillin	G418	This study
LentiCRISPRv2 Neo sgPTPN14-1	8391	221651	CATGACTGTCTC	U6	Ampicillin	G418	This study
LentiCRISPRv2 Neo sgPTPN14-3	8392	221652	CCACACTGGACG	U6	Ampicillin	G418	This study
