# Supplementary material for: HPV18 E7 inhibits LATS1 kinase and activates YAP1 by degrading PTPN14
Source: bioRxiv. 2024 Jun 19:2024.03.07.583953. Originally published 2024 Mar 7. Preprint. [Version 2] doi: 10.1101/2024.03.07.583953 (PMC10942435; doi:10.1101/2024.03.07.583953)
Supplement: Supplement 3 — Supplementary Table 3. List of antibodies used in the study. [file media-3.pdf]

Target	Description	Company	Catalog #	Application	Dilution
Beta-Actin	Mouse anti-bActin	CST	3700S	Western Blot	1:1000
PTPN14	Rabbit anti-PTPN14	CST	13808S	Western Blot	1:1000
YAP1	Mouse anti-YAP1	CST	12395S	Western Blot	1:1000
YAP1 pS127	Rabbit anti-YAP1 pS127	CST	4911S	Western Blot	1:1000
LATS1	Mouse anti-LATS1	Proteintech	66569-1-IG	Western Blot	1:1000
LATS1 pT1079	Rabbit anti-LATS1 pT1079	CST	8654S	Western Blot	1:1000
NF2/Merlin	Rabbit anti-Merlin	CST	12888S	Western Blot	1:1000
NF2 pS518	Rabbit anti-Merlin pS518	CST	13281	Western Blot	1:1000
RB1	Mouse anti-RB1	CalBiochem	OP66	Western Blot	1:500
HA	Rat anti-HA HRP conjugate	Roche	12013819001	Western Blot	1:500
WWC1/Kibra	Rabbit anti-Kibra	CST	8774S	Western Blot	1:1000
Keratin 10	Mouse anti Cytokeratin 10	Santa Cruz	sc-52318	Western Blot	1:1000
Mouse IgG	Horse anti-Mouse HRP conjugate	CST	7076S	Western Blot	1:2000
Rabbit IgG	Horse anti-Rabbit HRP conjugate	CST	7074S	Western Blot	1:2000
Mouse IgG	Goat anti-Mouse IRDye 680LT	LI-COR	926-68020	Western Blot	1:5000
Rabbit IgG	Goat anti-Rabbit IRDye 800CW	LI-COR	926-32211	Western Blot	1:5000
