# Supplementary figures and images for: HPV18 E7 inhibits LATS1 kinase and activates YAP1 by degrading PTPN14
Source: bioRxiv. 2024 Jun 19:2024.03.07.583953. Originally published 2024 Mar 7. Preprint. [Version 2] doi: 10.1101/2024.03.07.583953 (PMC10942435; doi:10.1101/2024.03.07.583953)

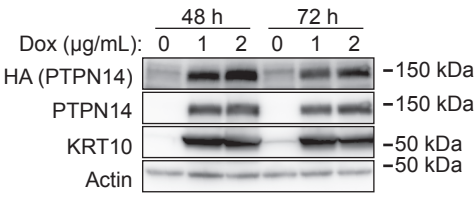

Supplement: Supplement 4 — Figure S1. PTPN14 increases KRT10 protein levels. N/Tert-1 Cas9:sgPTPN14 keratinocytes transduced with the pLIX-PTPN14 overexpression construct were incubated with 0, 1 or 2 μg/ml doxycycline for 48 hours or 72 hours. Whole cell protein lysates were separated by SDS-PAGE and proteins were detected by immunoblotting for HA, PTPN14, KRT10, and actin. [file media-4.pdf]

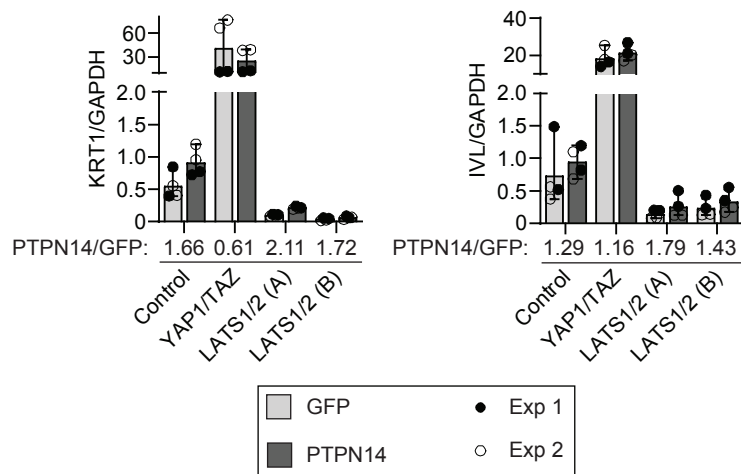

Supplement: Supplement 5 — Figure S2. PTPN14 increases KRT1 and IVL transcript levels. HFK transfected with siRNA for 72 hours were also transduced for 48 hours with lentiviral vectors for GFP or PTPN14 overexpression. Cells were transfected with siRNA: nontargeting control, YAP1, TAZ, and two separate pairs for LATS1 and LATS2 denoted (A) and (B). Total cellular RNA was measured by qRT-PCR for KRT1 and IVL for two biological replicate experiments, each performed in technical duplicate. Transcript levels were normalized to GAPDH. Graphs show mean ± standard deviation. Samples are the same as those analyzed in Figure 7. [file media-5.pdf]

A

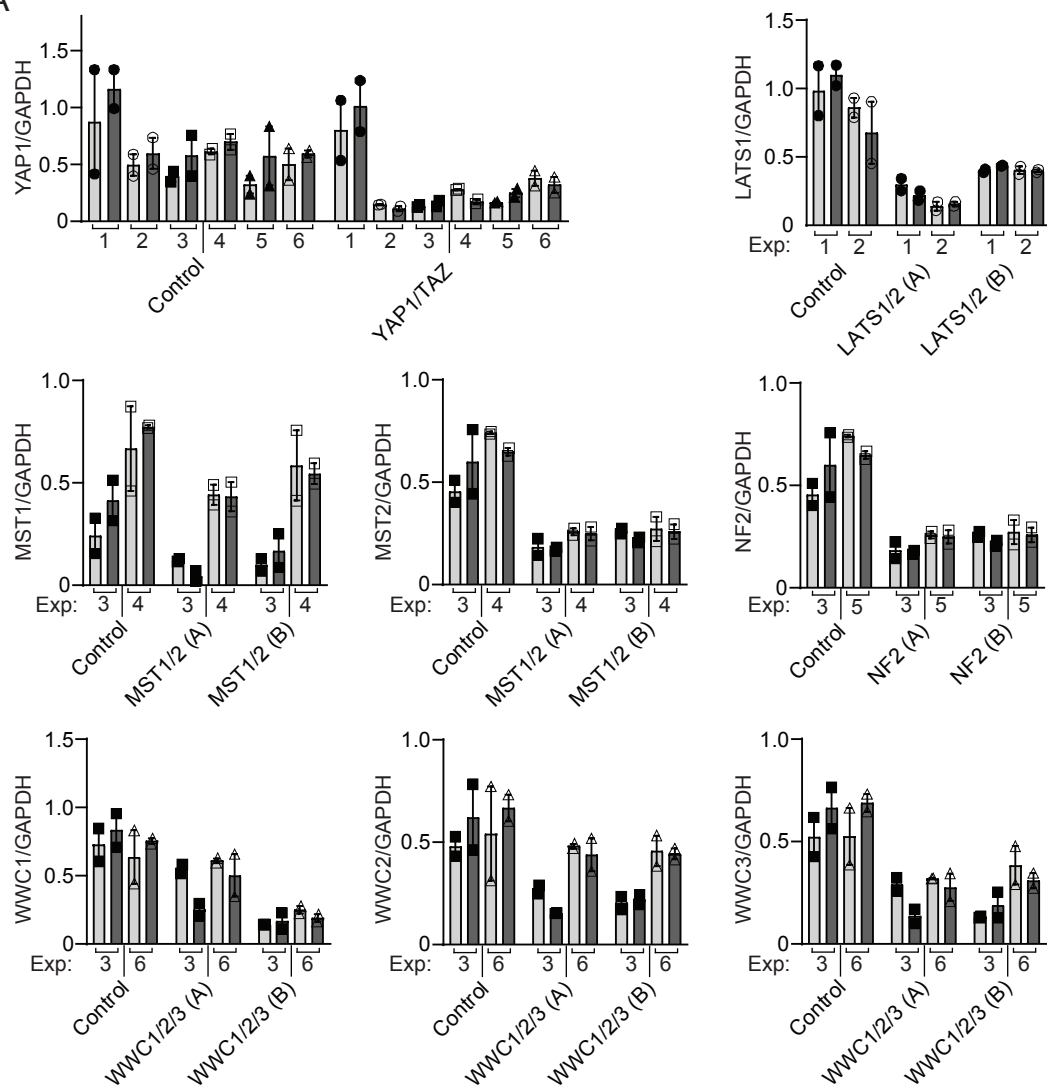

B

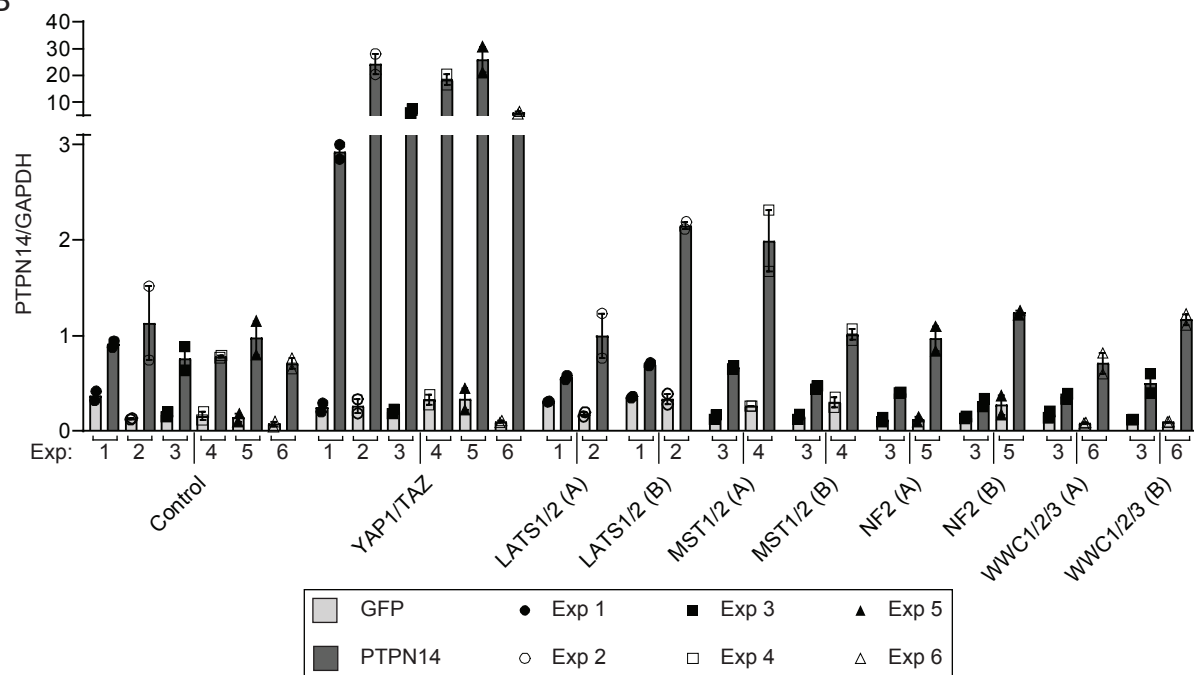

Supplement: Supplement 6 — Figure S3. Validation of siRNA knockdowns. HFK were transfected with siRNA then transduced with lentiviruses encoding GFP or PTPN14 at 24h post transfection. Total cellular RNA was collected 72h post-knockdown and 48h post-transduction. RNA transcripts for KRT10 were measured by qRT-PCR and normalized to GAPDH. Six individual experiments were conducted, each in technical duplicate, and samples are those that are analyzed in Figure 7. (A) Total cellular RNA was analyzed by qRT-PCR with primer sets as indicated to validate knockdown efficiency. Transcript levels are normalized to GAPDH. Graphs show mean RNA levels ± range. TAZ and LATS2 transcript levels were below the limit of detection and are not shown. (B) PTPN14 transcripts were measured in all six experiments by qRT-PCR, normalized to GAPDH, and plotted as mean ± range. [file media-6.pdf]

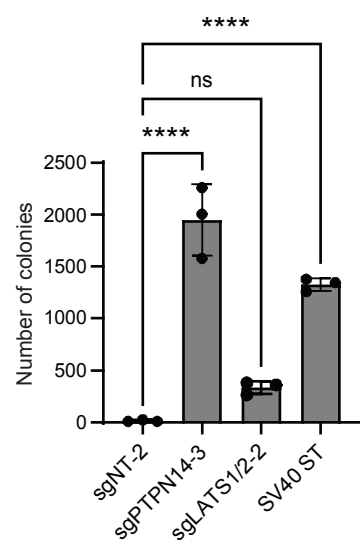

Supplement: Supplement 7 — Figure S4. PTPN14 knockout promotes anchorage independent growth and suppresses YAP1 phosphorylation in HEK TER cells. HEK TER cells expressing Cas9 were transfected with a second set of sgRNA compared to those used in Figure 8. HEK TER cells expressing SV40 ST were used as a positive control for colony formation. Cells were plated in soft agar in technical triplicates and incubated at 37°C for 18 days, then photographed. Colonies were counted and quantified using ImageJ software. Graphs show individual data points for each plate and indicate mean ± standard deviation. Statistical significance of the nontargeting control condition compared to experimental conditions was determined by ANOVA with Dunnett’s multiple comparisons test (ns, not significant; ****, P < 0.0001). [file media-7.pdf]
